# Periodontal Treatment Practices and Referral Profile of General Dentists in Maharashtra: A Questionnaire-Based Survey

**DOI:** 10.7759/cureus.79111

**Published:** 2025-02-16

**Authors:** Saniya A Shikalgar, Sameer A Zope, Girish Suragimath, Siddhartha Varma, Apurva V Kale

**Affiliations:** 1 Department of Periodontology, School of Dental Sciences, Krishna Vishwa Vidyapeeth (Deemed to be University), Karad, IND

**Keywords:** general dental practitioner, graduates, non-surgical, postgraduates, referral, surgical

## Abstract

Introduction

General dentists are generally confident in non-surgical periodontal care but tend to refer more complex surgical cases to periodontists due to limited experience and resources. This study aims to evaluate the range of periodontal treatments offered by general dentists, the frequency of their referrals to periodontists, the criteria for referrals, and the factors influencing these decisions.

Methods

This cross-sectional, descriptive study employed a questionnaire to examine the periodontal procedures performed by general dentists in both the rural and urban areas of Maharashtra and their referral practices. A simple random sampling method was used to select the participants. The questionnaire was in the form of a Google Form and was distributed to both graduate and postgraduate general dental practitioners via WhatsApp or email. The collected data were analyzed using IBM SPSS Statistics for Windows, Version 24 (Released 2016; IBM Corp., Armonk, New York, United States). The statistical test used was the Chi-square test and a p-value of less than 0.05 was considered statistically significant.

Results

A total of 348 responses were collected, with 209 from graduate practitioners and 139 from postgraduate practitioners. The majority of general dentists performed non-surgical procedures; however, 85% did not perform surgical treatments. About 35% felt inadequately trained in periodontics, leading to referrals. Referral patterns for both surgical (p=0.84) and non-surgical procedures (p=0.131) Chi-square tests indicated no significant differences between the referrals of graduates and postgraduates. Non-surgical cases were referred by 11% of the graduates and 8% of the postgraduates, while surgical cases were referred by 32% and 34% of them, respectively.

Conclusion

The study concluded that general dentists are confident in performing non-surgical periodontal procedures but express reservations about undertaking periodontal and implant surgeries. The primary factors influencing referrals to periodontists include insufficient experience, lack of appropriate facilities, and limited access to specialists. This highlights the need for enhanced periodontal education and resources for general dentists to improve patient care.

## Introduction

The increasing global lifespan, fueled by advancements in medical science, has presented new challenges in oral healthcare, particularly in managing periodontal diseases. With an aging population, the prevalence and severity of periodontal conditions have escalated, demanding more complex treatments and a greater reliance on specialist referrals [1]. While innovations in periodontal examination, diagnosis, and treatment have been made, they have also enhanced communication between general dentists, specialists, and other healthcare providers, fostering more coordinated care.

However, disparities in care quality remain, with patients visiting periodontists in private practice or dental institutes receiving better care than those treated by general dental practitioners (GDPs) [2]. Despite the emphasis on education in periodontics in dental institutions, undergraduate training often lacks comprehensive exposure to specialties, creating gaps in treatment delivery [3]. Additional barriers such as socioeconomic challenges, patient reluctance, and the non-referral tendencies of GDPs worsen the issue, leaving many patients without appropriate care [4,5]. Moreover, the dynamics of GDP-periodontist referral relationships are underexplored, with significant variations influenced by non-clinical factors [6].

There is a scarcity of research on the patterns of periodontal care provided by GDPs and their referral practices to periodontists. Hence, this study evaluated the scope of periodontal treatments performed by GDPs, the frequency of referrals to periodontists, and the factors influencing their referral decisions.

## Materials and methods

Study design

This cross-sectional, descriptive questionnaire-based study was conducted among dental practitioners from Maharashtra. The questionnaire was distributed through WhatsApp and email to all graduate and postgraduate GDPs in Maharashtra. The questionnaire consisted of three domains: demographic data, treatment procedures, and reason for referral. The first part included general demographic questions, such as the respondent's qualifications, years of experience, and the location of their dental practice. The second part focused on non-surgical and surgical periodontal procedures, as well as peri-implant surgeries. Respondents are asked to indicate whether they performed these procedures themselves or referred patients to a periodontist for proper treatment protocol. The final section focused on the reasons for referral, highlighting why dentists chose to refer patients to periodontists and the criteria they considered when selecting a periodontist.

Sample size estimation

The sample size was estimated to be approximately 200 participants, based on an alpha error of 5% and a study power of 80%, using G*power software version 3.1.9.7 (Heinrich-Heine-Universität Düsseldorf, Düsseldorf, Germany), with a power of ±1.96 and a 95% confidence interval. A total of 348 complete and valid responses were received, exceeding the required sample size for statistical analysis.

Ethical approval

The ethical clearance was obtained from the university ethics committee of Krishna Vishwa Vidyapeeth (Protocol no: 002/2024-2025) before commencing the study.

Pre-validation and pre-testing of the questionnaire

A specially designed, closed-ended questionnaire (Google Forms, Google LLC, Mountain View, CA, USA) consisting of 18 questions was developed. It was pre-tested and validated by experts from the Krishna Vishwa Vidyapeeth protocol committee for content and face validity. A pilot study with 30 participants was conducted to assess reliability, with Cronbach’s alpha calculated to determine internal consistency and test-retest reliability was assessed over a two-week interval.

Distribution of the questionnaire

The questionnaires were distributed via WhatsApp and email using contact details obtained from local Indian Dental Association (IDA) branches in Maharashtra. The email addresses and phone numbers of registered dental practitioners were collected from official IDA membership databases with prior permission. Simple random sampling was applied to ensure proportional representation across different regions and practice levels. Participants received an email or a WhatsApp message containing a digital consent form and a secure questionnaire link. Only responses from individuals who completed both the consent form and the questionnaire were included in the statistical analysis.

Statistical analysis

The collected responses were organized in Microsoft Excel (Microsoft Corporation, Redmond, WA, USA) and analyzed using IBM SPSS Statistics for Windows, Version 24 (Released 2016; IBM Corp., Armonk, New York, United States). Basic data were presented as percentages. The Pearson Chi-square test was used to compare groups based on practice area, years of experience, and treatment procedures (surgical and non-surgical). A p-value of less than 0.05 was considered statistically significant.

## Results

A total of 348 responses were collected for the survey, with 139 (39%) responses from postgraduate practitioners and 209 (60%) from graduate practitioners. Participants completed the survey once, with no time restrictions, after providing informed consent and understanding the study's aim, while maintaining anonymity. Among the participants, 134 (38.7%) GDPs had less than two years of practice experience, and 223 (64%) practiced in urban areas. 

The study findings presented in Table 1 indicate that 95 (26%) professionals had more than five years of experience. However, the majority of participants in this study (39%) were early-career practitioners, with less than two years of experience.

**Table 1:** Demographic characteristics of the general dentists Demographic parameters considered years of practice, location of the practice, and educational status.

Parameters	Respondents n (%)
Years of practice	
Less than 2 years	134 (39%)
2–5 years	119 (35%)
More than 5 years	95 (26%)
Location of practice	
Urban	223 (64%)
Rural	125 (36%)
Educational status	
Graduate	209 (60%)
Postgraduate	139 (39%)

Additionally, 125 (36%) dentists practicing in rural areas reported limited access to specialized clinics. In contrast, nearly 223 (64%) participants were based in urban areas, with most postgraduate practitioners also situated in urban settings.

Table 2 outlines the referral patterns for various periodontal treatments, including non-surgical procedures. The majority of GDPs handled non-surgical treatments independently, with 267 (70%) performing scaling and root planing, and 193 (55.4%) providing splinting. More than half of them also performed occlusal adjustments and periodontal maintenance. However, the referral rate for the delivery of local antimicrobials was significantly higher and approached 52%.

**Table 2:** Profile of the non-surgical periodontal treatment followed by the general dentists

Treatment procedure	Self n (%)	Referral n (%)
Scaling and root planing	267 (70%)	81 (23.2%)
Splinting	193 (55.4%)	155 (45%)
Occlusal adjustment	216 (62.1%)	132 (38%)
Periodontal maintenance	204 (58.6%)	144 (41%)
Local antimicrobial drug delivery	167 (48%)	181 (52%)

Complex periodontal surgeries, such as gingivectomy, crown lengthening, frenectomy, periodontal flap procedures, and regenerative treatments, were typically referred to periodontists by GDPs. Among these, crown-lengthening procedures were referred less frequently than others, with more than half of the GDPs performing them in their own clinics. In contrast, 277 (74.5%) practitioners chose to refer frenectomy cases to a periodontist. The majority of GDPs referred cases involving regenerative procedures for hard and soft tissue (91.5%) and periodontal flap procedures (92.4%) to specialists. Additionally, out of the 348 GDPs surveyed in this study, 292 (93%) referred implant-related surgeries, which include implant placement and site augmentation, to periodontists. Notably, the referral rate for implant-related surgeries was the highest among all the procedures (93%; Table 3).

**Table 3:** Profile of surgical periodontal treatment followed by the general dentists

Periodontal Procedures	Self n (%)	Referral n (%)
Gingivectomy	85 (31.2%)	263 (68.8 %)
Crown lengthening procedure	263 (68.8%)	85 (31.2%)
Frenectomy	71 (25.5%)	277 (74.5 %)
Periodontal flap procedures	59 (7.6%)	289 (92.4%)
Hard tissue and soft tissue regenerative procedures	65 (8.5%)	283 (91.5%)
Implant-related surgery, implant placement, and implant site augmentation	56 (7%)	292 (93%)

Table 4 presents an overview of the distribution of respondents, comparing the referral patterns for non-surgical and surgical periodontal procedures between graduate and postgraduate practitioners. For non-surgical procedures, 37 (11%) of graduates and 28 (8%) of postgraduates referred cases to periodontists. Also 112 (32%) of graduates and 120 (34%) of postgraduates referred surgical cases to periodontist. For both non-surgical and surgical procedures, the chi-square tests revealed no statistically significant differences in referral rates between graduates and postgraduates (p-value 0.131 and p-value 0.84). In terms of their knowledge of periodontics, 264 (76.6%) respondents felt that their dental education adequately prepared them for periodontal therapy. Additionally, 330 (95.4%) participants expressed interest in attending educational programs to enhance their skills in periodontal surgery.

**Table 4:** Overall distribution of respondents for the referral of periodontal surgical procedures p<0.05: Level of statistical significance.

Periodontal procedures	Dental practitioner (n= 348)	Self n (%)	Referral n (%)	Chi-square	p value
Non-surgical	Graduate (n=166)	129 (37%)	37 (11%)	2.289	0.1312
	Postgraduates (n=182)	154 (44%)	28 (8%)		
Surgical	Graduate (n=166)	54 (16%)	112 (32%)	0.03	0.842
	Postgraduates (n=182)	62 (18%)	120 (34%)		

**Figure 1:**
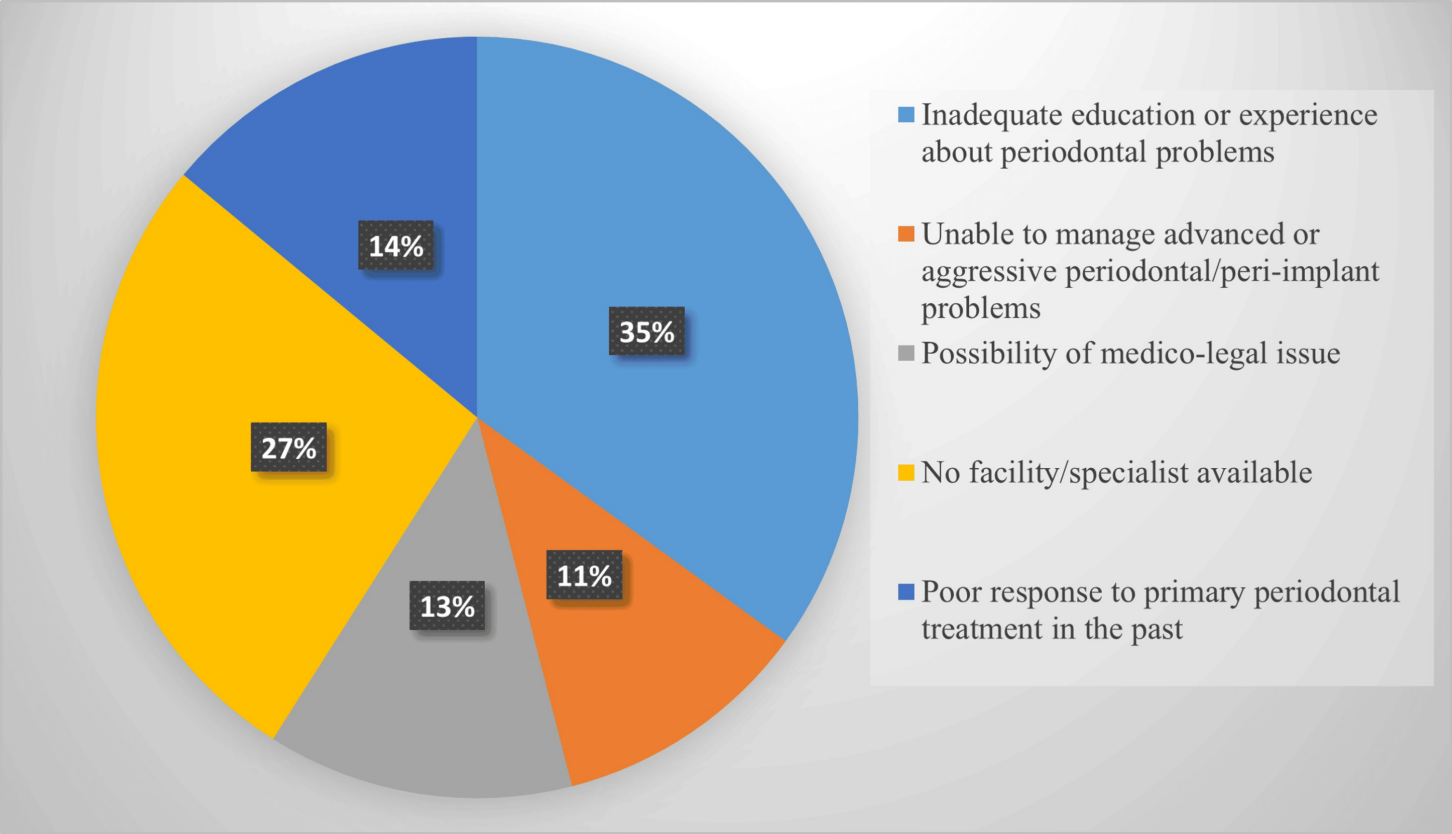
Reasons for referral from general dentists to periodontists

Figure 1 shows that 122 (35%) GDPs felt they had inadequate education and experience in periodontics. Moreover, 93 (27%) of them identified the absence of facilities and the unavailability of specialists in their area as the main reasons for referring patients to a periodontist for treatment.

**Figure 2:**
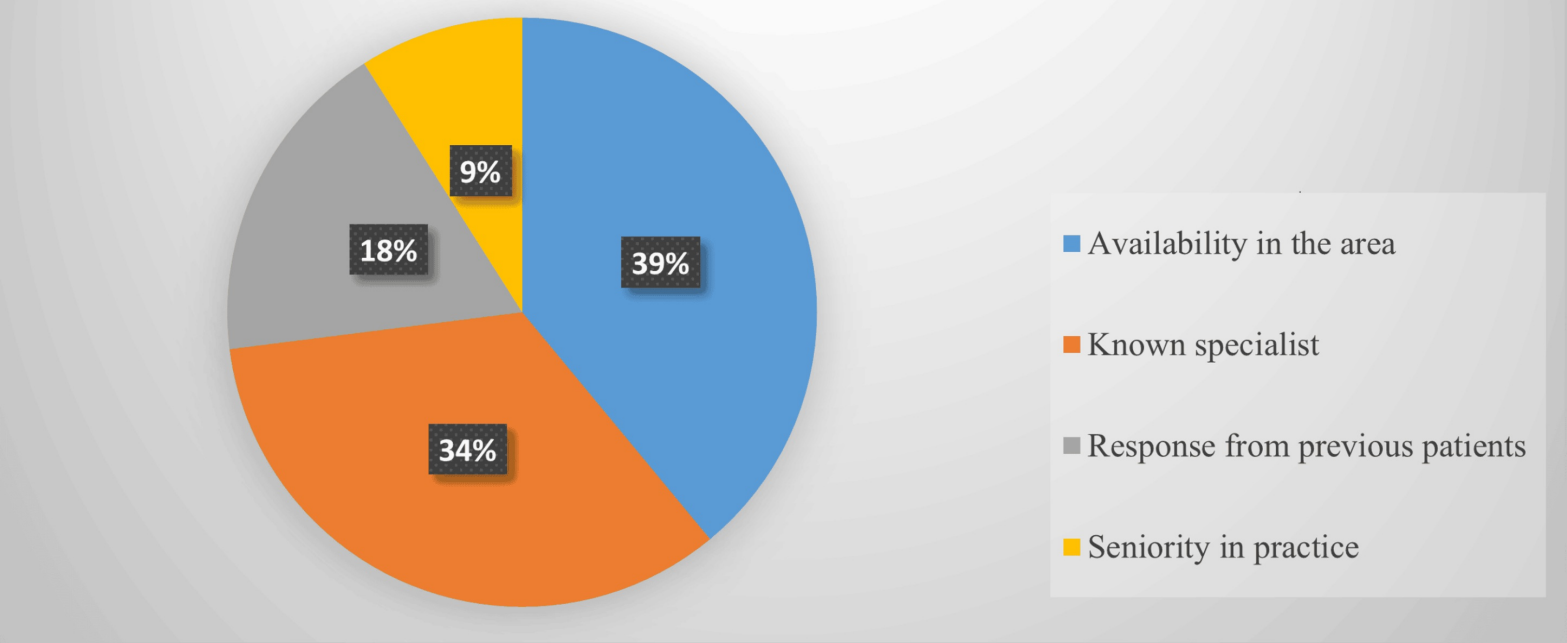
Criteria followed by general dentists in selecting a periodontist

The results showed that 135 (39%) GDPs referred patients to specialists located near their practices, while 118 (34%) were aware of specialists in their area. These factors significantly influenced the choice of the specialist (Figure 2).

## Discussion

GDPs are crucial for managing oral health, including periodontal diseases, but several factors limit their ability to provide optimal care. Referral to periodontists is a critical aspect of comprehensive dental care, particularly for cases involving complex periodontal diseases, implant placements, or advanced surgical procedures. Referral decisions are influenced by disease severity, confidence in managing complex cases, specialist availability, clinic limitations, patient motivation, and legal concerns. Despite the recognized importance of referrals in ensuring timely and appropriate care, there is a paucity of studies that specifically examine the referral practices and decision-making criteria employed by GDPs.

This study aimed to explore the periodontal treatment practices and referral patterns among GDPs in Maharashtra. A random sample of dentists was surveyed anonymously using a questionnaire. The survey collected information about their demographics, type of practice, treatments they offer, reasons for referrals, and the criteria they used when referring patients to periodontists. As dentists gain more experience, they tend to refer patients to specialists less often. Furthermore, the location of the dental practice plays a crucial role in shaping referral patterns. The results of the study also revealed that the years of experience and location of practice have a significant effect on the referral profile of the practicing dentists, whereas their educational qualifications had a significant influence on the referral profile of the practitioners. Compared to postgraduates, graduates had a high percentage of referral profiles for implant treatment.

Zemanovich et al. conducted a study to assess the demographic variables influencing patient referrals from general dental clinics to periodontists [2,3]. Their findings indicated that factors such as patient gender, the geographical location of the dental practice, and the proximity to a periodontist significantly impacted the referral patterns. Our study, conducted among GDPs in Maharashtra, yielded similar results, reinforcing these observations. Notably, most dentists in urban areas have better access to specialized clinics and referral options. The availability of periodontists and well-equipped dental facilities facilitates the referral of patients for advanced periodontal care. These findings emphasize the need to enhance specialist services in rural areas and ensure that all dentists, regardless of their location or experience, have access to the necessary resources to provide optimal periodontal care.

Our study findings are consistent with previous research by Jadhav et al. (2015) [7] and Mali et al. (2008) [8], which reported that 97% of dentists personally perform Phase-I therapy, often recommending mouthwashes and proper brushing techniques. Additionally, most GDPs carry out gingivectomy and crown lengthening procedures, while approximately 30% perform flap surgeries. In our study, we observed that surgical periodontal procedures are more frequently referred to periodontists, whereas non-surgical procedures were typically managed by the GDPs. Our results indicate that GDPs frequently perform crown lengthening procedures, consistent with a study by Ghiabi E et al., which found that 17% of GDPs reported independently conducting such procedures [9]. Furthermore, among periodontal surgical procedures, gingivectomy was the most commonly performed by GDPs, aligning with the findings by Rabi H et al. [10]. 

In our study, we identified four criteria for referring patients to a periodontist: inadequate education or experience, lack of facilities in the clinic, medicolegal issues, and the inability to manage advanced or aggressive periodontal or peri-implant problems. Among these, inadequate education or experience was found to be the most significant factor affecting referrals. Our findings are consistent with studies conducted by Ghiabi et al. and Rabi et al. [9,10]. Additionally, we highlighted four key factors in selecting a periodontist: the reputation of known specialists and the availability of specialists in the area. These results align with previous research conducted by Park et al. (2011) [4].

In the present study, there was no statistically significant difference found in groups for the referral profile of surgical and non-surgical periodontal surgeries. Very few practitioners performed all the periodontal surgical procedures by themselves. A majority revealed that they performed minor surgical procedures such as gingivectomy and frenectomy by themselves. However, a major surgical procedure such as ridge augmentation and soft tissue graft needed a referral to the periodontist. In our study, a majority of GDPs referred implant-related surgeries, implant maintenance. and management of peri-implant complications to periodontists [11,12].

Our study also did not indicate any statistically significant differences in the referral practices between graduate and postgraduate GDPs for both non-surgical and surgical procedures. This suggests that the level of academic qualification (graduate vs. postgraduate) does not significantly influence the decision to refer patients to periodontists. Also both groups recognized the importance of specialist intervention when necessary. This is consistent with the findings of Ismail et al. (2019), who reported that GDPs often refer periodontal cases to periodontists when the complexity of the case exceeds their comfort level or expertise, regardless of their additional qualifications [13].

The decision to refer a patient to a periodontist is influenced by multiple factors, including the complexity of the case, the confidence of the GDP in managing periodontal conditions, and the perceived benefits of specialist care. Bhati et al. (2016) emphasized that referral decisions are often driven by the practitioner's clinical experience and the perceived need for specialized care rather than their formal academic qualifications. This aligns with the current study's findings, where both graduate and postgraduate GDPs exhibited similar referral patterns, suggesting that clinical experience and case complexity may play a more significant role than academic training [14].

This survey revealed that most of the GDP felt that dental education programs had trained them well for performing periodontal surgeries. A majority of them showed more interest in attending educational programs/workshops to gain more information about periodontal treatments. These results have important implications for periodontal care and the relationship between GDPs and periodontists. This underscores the importance of fostering strong referral networks and communication between the two groups to ensure optimal patient outcomes.

Limitations and future perspective

While this study provides valuable insights into referral practices, it is important to acknowledge its limitations. It mainly focuses on one region, may have biases like social desirability and self-reporting bias, and lacks standard ways to evaluate periodontal practices and referrals. It mostly shows the views of GDPs, with little input from periodontists and patients. Since it is a cross-sectional study, it cannot track changes over time. Future research should include larger and more diverse groups, gather feedback from specialists, and look at how patient education and standardized referral processes affect care. Additionally, qualitative studies could provide deeper insights into the motivations and barriers behind the referral practices of GDPs to periodontists.

## Conclusions

In conclusion, this study indicated that GDPs are confident in performing non-surgical periodontal procedures, such as scaling, root planing, splinting, and maintenance. However, their confidence diminishes when it comes to periodontal surgical procedures, leading to a higher referral rate for complex treatments like regenerative procedures and implant surgeries. It also revealed that referral rates tend to decrease as practitioners gain more experience. Key factors influencing referral decisions include inadequate education and experience in managing periodontal issues, as well as the limited availability of facilities and specialists in rural areas. Experience of the GDP and the location of the practice were also significant contributors to the referral patterns. Additionally, many GDPs expressed a desire for more educational programs to improve their periodontal skills. The findings highlight the need for increased guidance and training to equip them better, ultimately enhancing patient referrals and treatment outcomes.
